# A Clinicopathological Study of Aural Polyps: A Retrospective Analysis in a Tertiary Care Hospital

**DOI:** 10.1055/s-0043-1777418

**Published:** 2024-04-12

**Authors:** Kothegala Chandrashekaraiah Prasad, Gopinath Indu Varsha, Kouser Mohammadi, Arjun Gupta, Diana Ann Jose

**Affiliations:** 1 Department of ENT and Head and Neck, Sri Devaraj Urs Academy of Higher Education and Research, Tamaka, Kolar, Karnataka, India

**Keywords:** suppurative otitis media, middle ear, temporal bone

## Abstract

**Introduction** Aural polyps are a misnomer. Any lesion can present as a mass in the external auditory canal. Aural polyps are proliferation of the granulation tissue due to long standing inflammatory process with associated otalgia and otorrhea.

**Objectives** To document the clinicoradiological presentations, intraoperative findings, and histopathological diagnosis of aural polyp, correlating them.

**Methods** In our study 81 patients underwent treatment for aural polyps in the department of Ear, Nose, and Throat (ENT) from April 1997 to April 2022. Results were tabulated, a simple descriptive analysis was done using the Statistical Package Social Sciences software, and the results obtained were represented as percentages and presented in tables.

**Results** The majority (38) of the patients presenting with aural polyps were diagnosed with mucosal and squamous type of CSOM, and 22 with simple granulation polyps. There were also 5 patients with malignant otitis externa, 3 patients had glomus tumors, 2 patients with retained foreign bodies, and 3 patients with brain herniation. We also identified aberrant internal carotid artery, high jugular bulb, one patient had facial nerve neuroma, one patient had polyp from the tragus diagnosed with tuberculosis, one patient with keratosis obturans, and one with exostosis.

**Conclusion** A thorough detailed examination and mastoid exploration with radiological and histopathological evaluation is mandatory for better defining the definitive treatment. Utmost care and meticulousness are advised for the surgeons while dealing with aural polyps to avoid any complications.

## Introduction

The ear can manifest a spectrum of disorders, including those from surrounding structures. Ear diseases, unless extensive with outward complications, can cause morbidity and affect patients' quality of life. In this study, we will discuss the disorders manifested as aural polyps at our tertiary care center.

Aural polyps are a misnomer. Any lesions, irrespective of the underlying pathology, can present as a mass in the external auditory canal but can also arise from the middle ear. It is commonly defined as a soft to rubbery, pale pink to reddish mass that presents in the external auditory canal. 1 Usually, aural polyps will be a proliferation of the granulation tissue, in response to long-standing inflammatory process, presenting as mass in the ear canal with associated otalgia and otorrhea. 2

According to the literature, approximately 55% of the aural polyps happen due to an underlying inflammatory pathology, followed by presentations associated with mucosal or squamosal chronic suppurative otitis media. 2 The most common site of origin is the middle ear cleft, but they can also arise from the external auditory canal, facial nerve, glomus bodies, parotids, and even metastatic lesions. 2

In our study, we documented the manifestations of disorders presented as aural polyps. Hence, obtaining a clinical, radiological, and histopathological diagnosis is mandatory in all cases of aural polyps. To avoid compromising the definitive treatment, it is advisable to explore the mastoid under general anesthesia to better understand the pathology and extent, followed by histopathological confirmation of the diagnosis.

## Methods

### Clinical Data

In our institution, 81 patients underwent treatment for aural polyps in the ear, nose, and throat (ENT) department from April 1997 to April 2022. The clinical details, pure-tone audiometry, radiological, and histopathology details of the 81 patients diagnosed with aural polyps were retrospectively analyzed from the medical records section of the department. An institutional ethics committee clearance was obtained for this study.

Results were tabulated, a simple descriptive analysis was done using the Statistical Package Social Sciences (SPSS) software, and the results obtained were represented as percentages and presented in tables.

## Results

Out of the 81 patients presented with aural polyps, 63% were males, age ranging from 7 to 60-years-old, and with involvement of right ear more than the left as shown in Tables 1 and 2 .

**Table 1:** Demographic features and symptoms

	N = 81(%)
Males	51 (63%)
Females	30 (37%)
Right ear	48 (59%)
Left ear	33 (41%)
Ear discharge	75 (93%)
Hearing loss/blocking sensation	78 (96%)
Tinnitus	28 (35%)
Ear pain	17 (21%)
Facial nerve palsy	05 (6%)
Hypoglossal nerve palsy	01 (1%)

**Table 2:** Diagnosis postexamination

Sl No.	Diagnosis	N = 81(%)
1.	Chronic suppurative otitis media	38 (47%)
2.	Granulation polyp	22 (27%)
3.	Malignant otitis externa	5 (6%)
4.	Glomus tumours	3 (4%)
5.	Neglected foreign body	2 (2%)
6.	Aberrant internal carotid artery	1 (1%)
7.	High jugular bulb	1 (1%)
8.	Tuberculosis	1 (1%)
9.	Brain herniation	3 (4%)
10.	Keratosis obturans	1 (1%)
11.	Exostosis	3 (4%)
12.	Facial neuroma	1 (1%)

**Fig. 1:**
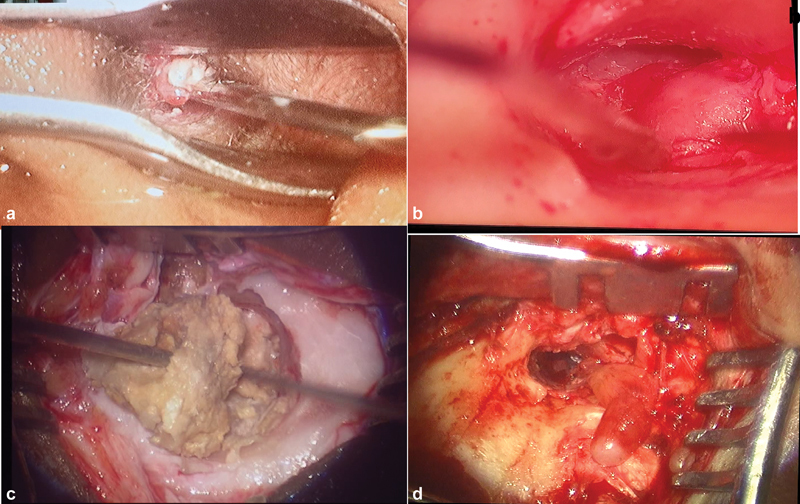
a) Image showing aural polyp with Cholesteatoma, b) aural polyp before elevation of the Tympanic membrane, c) Cholesteatoma debris, d) polyp arising from the retrotympanum with a thin stalk attachment and broad base.

**Fig. 2:**
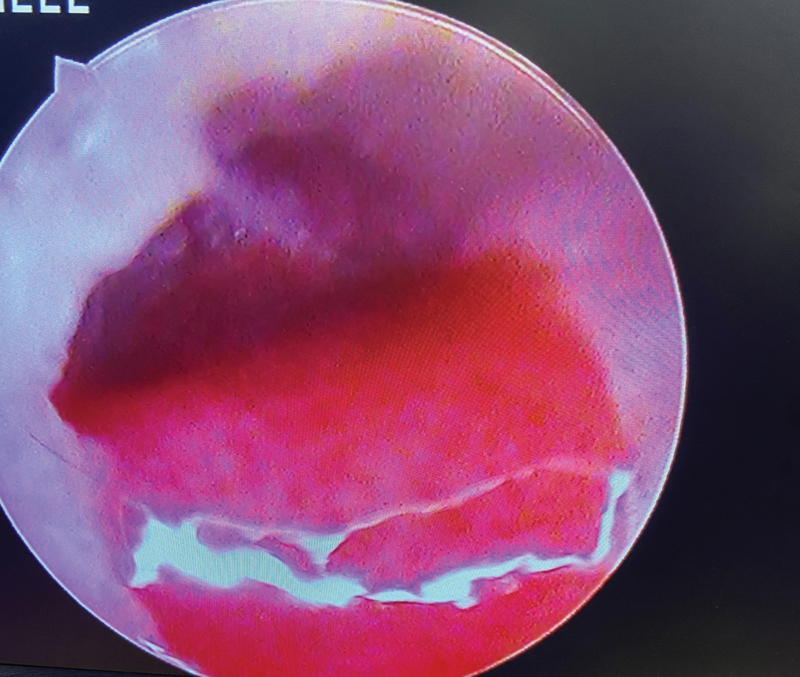
Image showing Granulation polyp.

All patients underwent a detailed ENT examination, with audiological and radiological evaluations. Among the 81 patients, 38 had complaints of foul-smelling, occasional blood-tinged otorrhoea, along with abnormal mass in the external auditory canal. Radiological and histopathological findings were consistent with squamous type and mucosal type of chronic suppurative otitis media. The polyp was attached to the retrotympanum through a thin stalk with a broad base. All patients underwent modified radical mastoidectomy, temporalis fascia grafting, and ossicular reconstruction. One patient with extensive cholesteatoma underwent surgical labyrinthectomy, blind sac closure, and canal wall mastoidectomy ( Fig. 1 ) In 22 patients, histopathology revealed as simple granulation polyp ( Fig. 2 ).

**Fig. 3:**
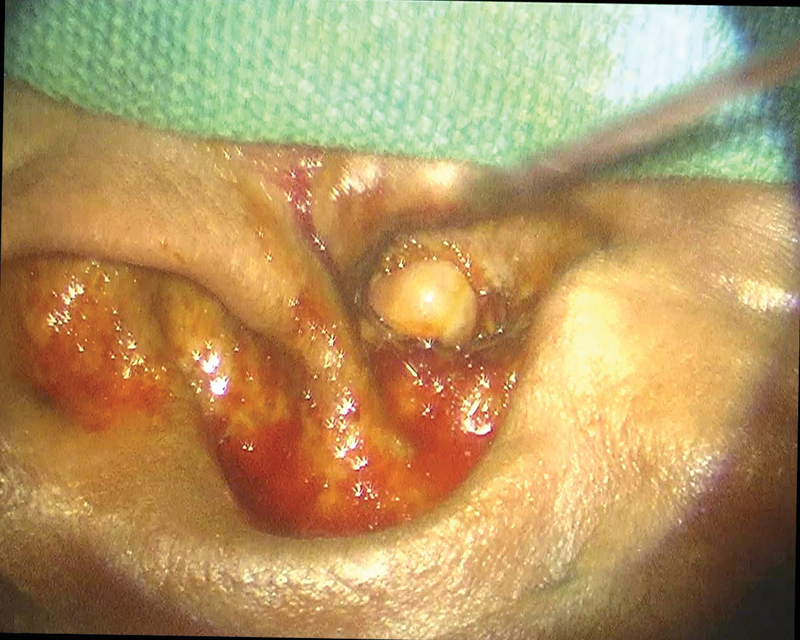
Image showing aural polyp in Malignant Otitis externa.

From the original group of 81, there were 5 patients who were diagnosed with malignant otitis externa, all of them with type II diabetes mellitus. Among which 2 patients presented with facial nerve palsy, one of which had facial canal dehiscence and high jugular bulb up to the round window. One patient had isolated hypoglossal nerve palsy, along with the presentation of an aural polyp. One patient had infection extending to the greater wing of the sphenoid and facial nerve palsy. All the patients underwent mastoid exploration with the clearance of disease, along with intravenous antibiotics ( Fig. 3 ).

**Fig. 4:**
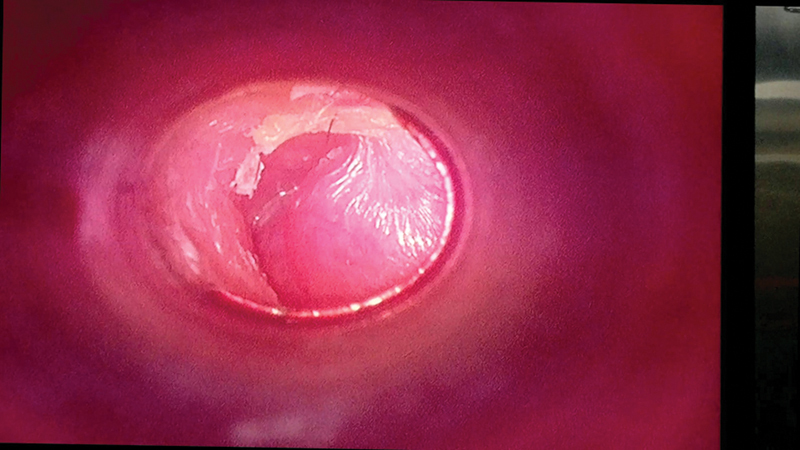
Image showing aural polyp in Glomus Tumour.

There were 3 elderly females who presented with a pulsatile mass in the external auditory canal, occasional blood-stained discharge, and tinnitus. They were diagnosed with glomus tumor clinically and radiologically ( Fig. 4 ). One patient defaulted treatment and the other two underwent excision and reconstruction. An 8-year-old boy presented with a post-aural abscess and abnormal mass in the external auditory canal. A 32-year-old male patient had an abnormal mass in the external auditory canal and foul-smelling discharge. A high-resolution computed tomography (CT) scan of the temporal bone showed soft tissue enhancement in both the patients. They were subsequently prepared for emergency mastoid exploration and surgical procedure. Intraoperatively, neglected foreign bodies were found: the boy had a piece of stick that formed a postauricular fistula with an abscess, and the older male patient had two insects behind the external auditory canal polyp. The tympanic membrane and middle ear cavity were normal.

**Fig. 5:**
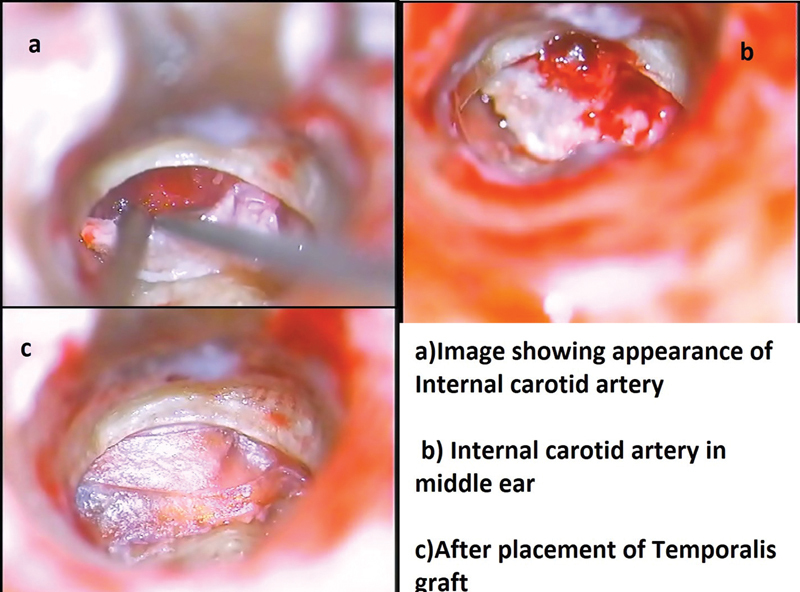
5a) Image showing appearance of Internal carotid Artery, 5b) Internal carotid artery in middle ear, 5c) after placement of Temporalis graft.

Vascular lesions presented as aural polyps in the external auditory canal in two patients: one with an aberrant internal carotid artery, as a vascular loop, and one with a high jugular bulb with dehiscence of the external auditory canal's floor ( Fig. 5 ). The patients presented with symptoms of hearing loss and tinnitus. They underwent canal-wall-down mastoidectomy and reconstruction. Temporalis fascia grafting was done covering the internal carotid artery intraoperatively.

**Fig. 6:**
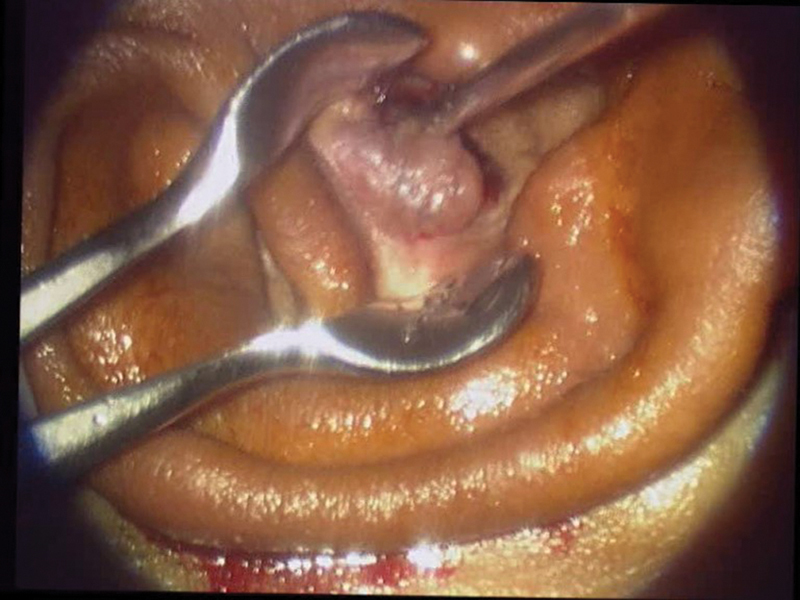
Image showing aural polyp in Tuberculosis.

An elderly female with type II diabetes mellitus presented with an abnormal mass in the external auditory canal with severe otalgia and trismus. On examination, her cartilage of the external auditory canal was eroded. She was posted for emergency mastoid exploration after a high-resolution CT scan of the temporal bone. The radiology revealed cartilage necrosis and an abnormal mass confined to the external auditory canal. Intraoperatively, under general anesthesia, the necrosed mass was excised. Tympanic membrane and middle ear were normal. The histopathology findings evidenced tuberculosis, and treatment began with prophylactic ATT. On follow-up, she had stenosis of the external auditory canal ( Fig. 6 ).

**Fig. 7:**
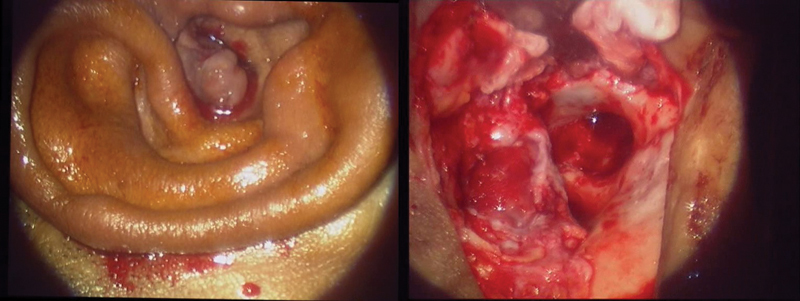
Image showing aural polyp in brain herniation with the tegmen tympani defect.

Furthermore, 3 patients presented with aural polyp; the radiological investigation showed brain herniation with the tegmen tympani defect ( Fig. 7 ). All of them underwent mastoid exploration. One patient had multiple defects with CSF otorrhoea and underwent blind sac closure. Another one underwent cranialization of the mastoid cavity. As for the third patient, the defect was reconstructed with septal cartilage. All patients were put on higher doses of intravenous antibiotics (IV) antibiotics pre- and postoperatively. Moreover, there was one case of keratosis obturans, and the patient underwent mastoid exploration with post auricular soft tissue obliteration of the cavity and skin grafting for tympanomeatal flap; they are now on regular follow-up. There were also three patients were diagnosed with exostosis of the external auditory canal and underwent excision.

**Fig. 8:**
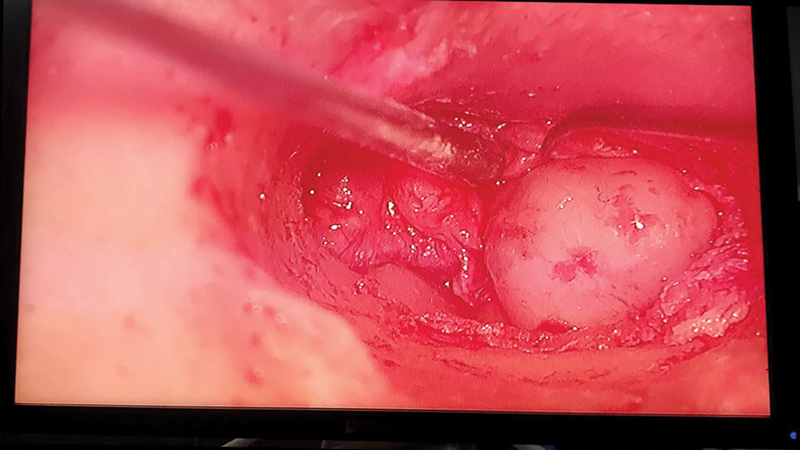
Image showing aural polyp in Facial Neuroma.

Finally, a 65-years-old female patient presented with grade II House–Brackman facial nerve palsy, otorrhea, and aural polyp. Radiological investigation revealed a mass arising from the facial nerve. On mastoid exploration, mass was excised from the facial nerve followed by grafting was done. The mass was sent for histopathology, revealed as facial neuroma. Patient is on regular follow-up and facial nerve palsy has improved ( Fig. 8 ).

## Discussion

In our study, 81 patients were diagnosed with aural polyps from April 1997 to April 2022. The majority were male, with involvement of right ear being more frequent than of the left. Among our cohort, 46% had associated cholesteatoma and 27% had simple granulation polyp, followed by malignant otitis externa, brain herniation, glomus tumors, foreign bodies, tuberculosis, keratosis obturans, and exostosis of the external auditory canal. The main presenting complaints were feeling of blockage and foul-smelling otorrhoea, followed by tinnitus and otalgia. Preoperatively, all patients underwent a detailed ENT examination, as well as audiological and radiological evaluations. The anatomy of the ear with anomalies and the different pathologies have been noted, and all patients underwent mastoid exploration and reconstruction. The mass in the external auditory canal was sent for histopathological evaluation, and the reports were documented.

Aural polyp is considered a misnomer. Due to the high prevalence of chronic suppurative otitis media, with the common clinical presentations like foul-smelling ear discharge, and difficulty hearing, patients with sinister lesions can get inadvertently misdiagnosed, leading to improper treatment. A preoperative biopsy cannot establish a correct diagnosis since it shows only a peripheral part of the underlying disorder and can even lead to complications, in the case of vascular lesions. Hence, a detailed, thorough ENT and radiological evaluation is mandatory. A high resolution CT scan of the temporal bone is the preferred radiological imaging to understand the complexities and to proceed with the management.

According to the literature, polyps related to chronic suppurative otitis media and due to underlying inflammatory pathology are common or secondary to pulmonary or other systemic tuberculosis. 3 Studies have estimated the incidence of granulomatous diseases as 3 to 5% in the surgical specimens. 4 Furthermore, there are documented cases of tuberculosis presentation with multiple tympanic membrane perforations, as well as limited presentations of tuberculous otitis media. The aberrant course of the internal carotid artery in the middle ear is also known; 5 in our study, the artery presented as a vascular loop, and after mastoidectomy it has been covered with temporalis fascia grafting. With dehiscence of the floor of the middle ear wall, the high jugular bulb can be seen even up to the level of the stapes supra structure. 6 However, there are no studies in literature is quoted on Tuberculosis presenting as aural polyp.

Tegmen erosion can happen secondary to any extensive squamous disease, due to underlying inflammatory or malignant pathology, trauma, and/or brain herniation 7 9 10 . In our study, the brain herniation presented as an aural polyp. In one case, blind sac closure was done; in another, reconstruction of the defect and cranialization of the mastoid cavity were done. In the modern era, radiological evaluations and innovations to navigating surgeries play a pivotal role—especially among the surgeons. 8 A thorough visualization of the normal vital structures with their deviations and disease progressions is vital prior to any extensive surgeries. In the majority of our cases, radiological evaluation was documented as soft tissue attenuation with hyperintense or hypointense areas involving the external auditory canal, middle ear, and mastoid cavity.

Few publications have documented the importance of preoperative biopsy for a definitive diagnosis, but we do not recommend it without a better understanding of the underlying pathology. Polyps must be explored in a setting with available facilities to tackle any complications. When intraoperatively accessing the polyps, especially from the retro tympanum, utmost care is required. Mass lesions can never be pulled, so it is important to avoid using sharp instruments near them while elevating the tympanomeatal flap. Avoiding the fibrous annulus is also recommended in case of vascular lesions.

In cases of presentations of aural polyps, medical professionals must rely on a thorough detailed clinical history, clinical examination including any surrounding structures in case of extension, as well as underlying inflammatory processes. An in-depth radiological evaluation is needed prior to surgery. Utmost care and meticulousness are advised while working with aural polyps, and histopathological tissue diagnosis postoperatively is mandatory so as not to compromise the patients' definitive treatment.

## Conclusion

Aural polyps are a misnomer. They can manifest as symptoms within the ear per se or in the surrounding structures. A thorough, detailed examination and mastoid exploration, along with radiological and histopathological evaluation, are mandatory for the sake of treatment. Utmost care and meticulousness are advised for surgeons when dealing with aural polyps, to avoid any complications.
